# Short-Term Antibiotic Treatment Has Differing Long-Term Impacts on the Human Throat and Gut Microbiome

**DOI:** 10.1371/journal.pone.0009836

**Published:** 2010-03-24

**Authors:** Hedvig E. Jakobsson, Cecilia Jernberg, Anders F. Andersson, Maria Sjölund-Karlsson, Janet K. Jansson, Lars Engstrand

**Affiliations:** 1 Department of Bacteriology, Swedish Institute for Infectious Disease Control, Solna, Sweden; 2 Department of Microbiology, Tumor and Cell Biology, Karolinska Institute, Stockholm, Sweden; 3 Limnology/Department of Ecology and Evolution, Evolutionary Biology Centre, Uppsala University, Uppsala, Sweden; 4 Department of Microbiology, Swedish University of Agricultural Sciences, Uppsala, Sweden; 5 Ecology Department, Lawrence Berkeley National Laboratory, Berkeley, California, United States of America; Columbia University, United States of America

## Abstract

Antibiotic administration is the standard treatment for the bacterium *Helicobacter pylori*, the main causative agent of peptic ulcer disease and gastric cancer. However, the long-term consequences of this treatment on the human indigenous microbiota are relatively unexplored. Here we studied short- and long-term effects of clarithromycin and metronidazole treatment, a commonly used therapy regimen against *H. pylori*, on the indigenous microbiota in the throat and in the lower intestine. The bacterial compositions in samples collected over a four-year period were monitored by analyzing the 16S rRNA gene using 454-based pyrosequencing and terminal-restriction fragment length polymorphism (T-RFLP). While the microbial communities of untreated control subjects were relatively stable over time, dramatic shifts were observed one week after antibiotic treatment with reduced bacterial diversity in all treated subjects in both locations. While the microbiota of the different subjects responded uniquely to the antibiotic treatment some general trends could be observed; such as a dramatic decline in Actinobacteria in both throat and feces immediately after treatment. Although the diversity of the microbiota subsequently recovered to resemble the pre treatment states, the microbiota remained perturbed in some cases for up to four years post treatment. In addition, four years after treatment high levels of the macrolide resistance gene *erm*(B) were found, indicating that antibiotic resistance, once selected for, can persist for longer periods of time than previously recognized. This highlights the importance of a restrictive antibiotic usage in order to prevent subsequent treatment failure and potential spread of antibiotic resistance.

## Introduction

Within each anatomical niche in the human body, a complex, specialized microbiota is found [1]. The normal intestinal microbiota has several beneficial roles including protection against pathogen invasion, development of the immune system, and in nutrition [2], [3], [4], [5], [6]. Several external factors can cause disturbances or alter the bacterial composition. For example, antimicrobial treatment is known to cause short-term changes in the composition of the normal human microbiota [7], but also long-term consequences have been shown [8], [9], [10], [11]. One concern with the administration of antibiotics is the possibility of selection of antibiotic resistant strains of bacteria; not only in those which the antibiotic is directed towards but also among the normal microbiota [10]. The human indigenous microbiota could thereby potentially serve as a reservoir of resistance genes and contribute towards antibiotic resistance development [12], [13].

A commonly used treatment regimen for *Helicobacter pylori*, a Gram negative rod colonizing the gastric mucosa, is a triple therapy with clarithromycin, metronidazole, and omeprazole [14], [15], [16]. This treatment can lead to antibiotic resistance development among *H. pylori* strains [16] and also among members of the normal microbiota [7], [9], [10], [11], [17]. One mechanism for macrolide resistance is via erythromycin resistance methylases encoded by *erm* genes. These genes have been found in different genera with *erm*(B) having the largest host range [18]. The *erm*(B) gene is normally found on transposons located in the chromosome or on plasmids [18] and encodes a ribosomal methylase that methylates the 23S ribosomal RNA and thereby prevents the antibiotic from binding [19].

A few reports [7], [20], [21] have described microbiological changes following an eradication therapy containing clarithromycin, metronidazole and omeprazole using traditional culture-based methods. However, recent studies based on 16S rRNA gene analysis have shown that about 60–80% of the bacterial community in the intestine has not yet been cultured, in part because of unknown growth requirements [22]. Therefore, the impact of this commonly used antibiotic treatment therapy on the majority of the human microbiota is not fully known, especially not the long-term consequences.

Different molecular approaches have been applied to directly assess the diversity and composition of human-associated bacterial communities without the necessity for cultivation [22], [23], [24], [25]. Most studies have focused on the gut environment, although an increasing number of body sites are being investigated [1]. For example, recently a highly diverse microbiota was found in the throat [24] with 152 different phylotypes. The healthy microbiota has also been characterized in the distal esophagus [26] and stomach [27]. The largest number of bacteria is found in the colon [25], where it has been estimated that approximately 10^11^ bacteria colonize 1 gram of feces [28].

One example of a DNA-based community fingerprinting approach that has been used to study antibiotic effects on the gut microbiota is terminal restriction fragment length polymorphism (T-RFLP) [29]. We previously used this approach to monitor the impact of clindamycin administration on the fecal microbiota [8]. Both short-term community shifts and long-term impacts (i.e. 2 years) on the *Bacteroides* populations were observed.

In the present study, we used a combination of molecular approaches to assess the impact of a commonly used antibiotic treatment for *H. pylori* on the bacterial community composition in throat and stool samples over a four-year time period. By using 16S rRNA gene pyrosequencing and T-RFLP we aimed to determine whether antibiotic treatment resulted in long term shifts in the microbial community structure in the samples. In addition, we used real-time PCR to monitor *erm*(B) gene resistance levels up to four years after treatment.

## Results

### Long-term stability of the indigenous microbiota

The T-RFLP and pyrosequencing results were highly complementary. T-RFLP provided a rapid overview of changes in dominant members of the community, whereas the pyrosequencing data provided in depth information about changes in relative amounts of thousands of OTUs. Several thousand pyrosequencing reads were analyzed in all patients and controls and approximately 5000–11000 pyrosequencing reads in either throat or feces were analyzed per subject (Table S1).

The T-RFLP data were highly reproducible based on replicate extractions from the fecal samples and restriction digestions with three different restriction enzymes (data not shown). Overall the T-RFLP results correlated well with the 454-pyrosequencing results regarding the most dominant members of the microbiota. The most dominant TRFs (HaeIII digestion; 272–276 bp) in the fecal community were members of the *Clostridium coccoides* subgroup within the Firmicutes phylum, according to the RDP database (Figures S1, S2, and S3). The relative abundances of these TRFs were positively affected in patient D and negatively affected in patients E and F. In addition, the 285-bp TRF, corresponding to *Enterococcus faecalis* according to the RDP database, became very dominant in patients E and F immediately after treatment and represented more than 20% of the fecal microbiota (Figures S2 and S3).

**Figure 1:**
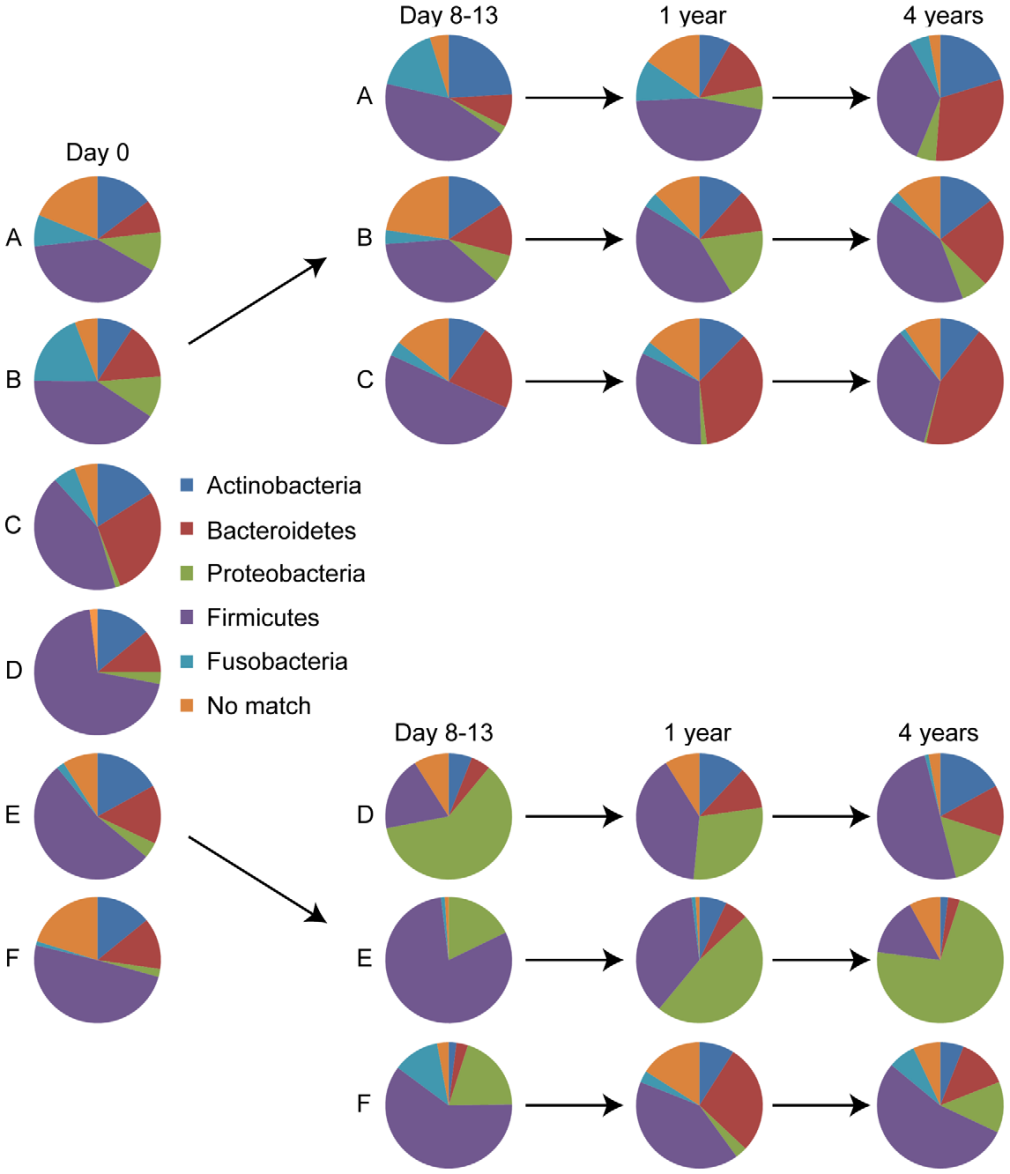
Phyla distribution in throat samples. Pie charts showing the phyla found in throat samples at day 0, day 8–13, 1 and 4 years in three controls (A, B, and C) and three antibiotic treated patients (D, E, and F). By using 16S rRNA pyrosequencing five different phyla were found in the throat samples; Firmicutes, Proteobacteria, Actinobacteria, Bacteroidetes, and Fusobacteria.

**Figure 2:**
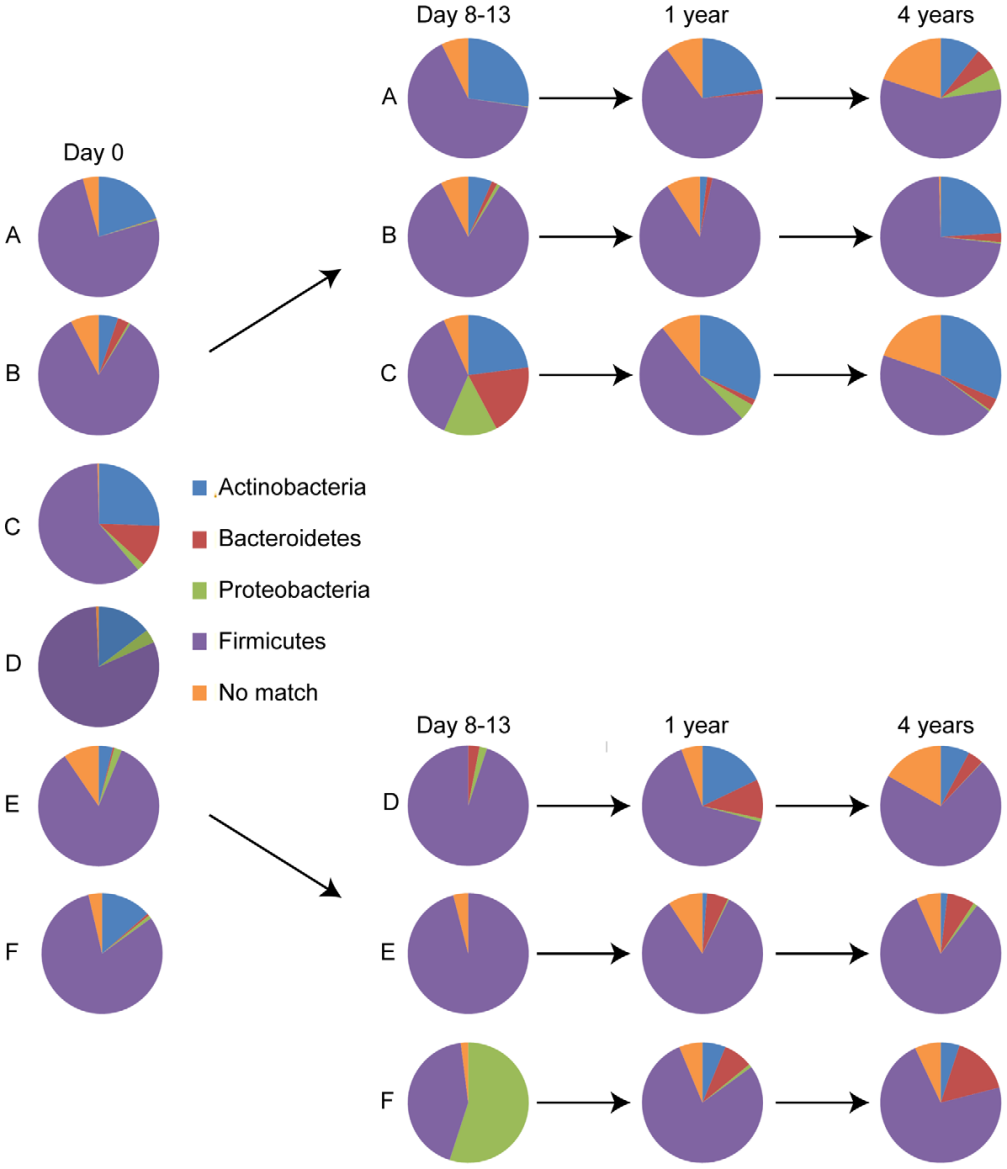
Phyla distribution in fecal samples. Pie charts showing the phyla found in fecal samples at day 0, day 8–13, 1 and 4 years in three controls (A, B, and C) and three antibiotic treated patients (D, E, and F). By using 16S rRNA pyrosequencing four different phyla were found in fecal samples; Firmicutes, Proteobacteria, Actinobacteria, and Bacteroidetes.

**Figure 3:**
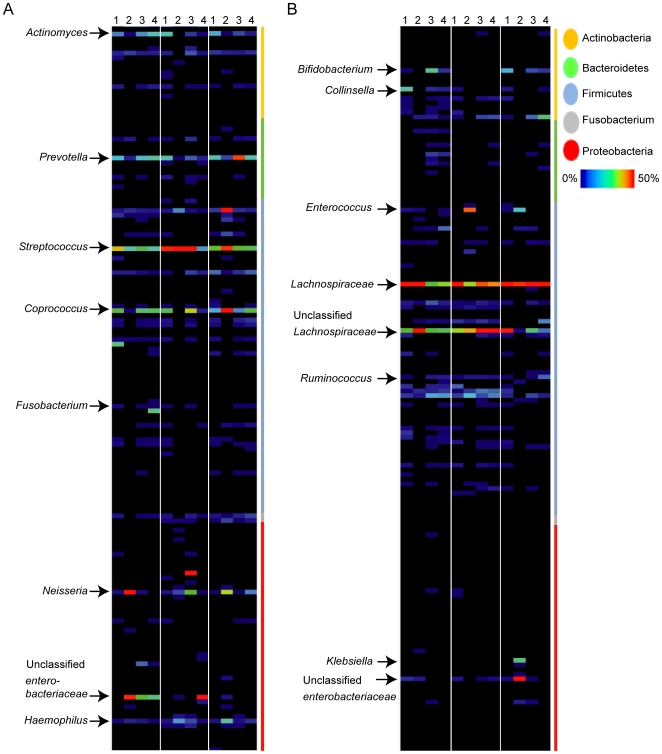
Individualized antibiotic responses. The heat maps show the relative abundance per sample of different taxonomic groups found in throat (A) or fecal (B) patient samples. The color panel shows the percent relative abundance (0–70%) of different taxonomic groups within the major phyla (Actinobacteria, Bacteroidetes, Firmicutes, Fusobacterium and Proteobacteria) detected from patients at day 0 (1), day 8–13 (2), 1 year (3) and 4 years (4) after treatment.

For analysis of the pyrosequencing data, the relative amounts of the different phyla were first determined in the control group and these amounts were used as a baseline for reference to subjects in the treated group. The throat microbiota in all six samples was dominated by five bacterial phyla; Firmicutes, representing 49% of the pyrosequencing reads (mean value of the six individuals, for individual values see Table S2), followed by Bacteroidetes (15%), Actinobacteria (14%), Fusobacteria (6%) and Proteobacteria (5%) (Fig. 1). The average fecal microbiota in all six samples was dominated by four bacterial phyla; Firmicutes (78%) followed by Actinobacteria (14%), Bacteroidetes (3%) and Proteobacteria (2%) (Fig. 2; for individual values see Table S3). Overall, the most dominant taxonomic groups at the genus level found in throat samples were e.g. *Streptococcus*, *Prevotella*, *Coprococcus*, *Actinomyces,* and *Neisseria* (Fig. 3A, Fig. S4A and Table S4). In fecal samples, the most dominant taxonomic groups were e.g. *Lachnospiraceae Incertae Sedis*, unclassified *Lachnospiraceae*, *Bifidobacterium*, *Collinsella*, unclassified *Ruminococcaceae* (Fig. 3B, Fig. S4B, Table S4).

**Figure 4:**
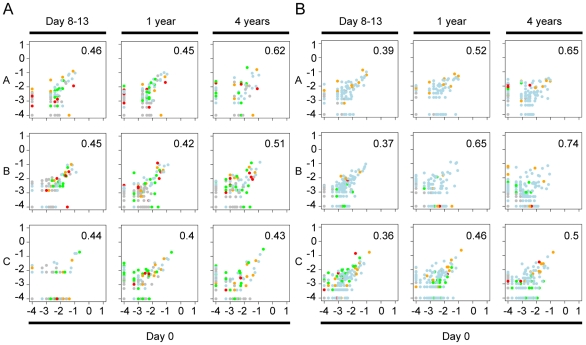
Correlation plots for the controls. Correlation plots showing OTU frequency at day 0 (x-axis), and day 8–13, 1 and 4 years (y-axis) in throat (A) and fecal (B) samples in the controls (A, B, and C). Bray-Curtis values are indicated as numbers in the figure as a number. A Bray Curtis value of 0 suggest the two sites have the same composition and 1 means the two sites do not share any species. The color of the dots represent different phyla: yellow, Actinobacteria; green, Bacteroidetes; blue, Firmicutes; red, Proteobacteria; grey, other phyla. Percentages of inter-sample variation explained by the two axes are shown in the figures.

**Figure 5:**
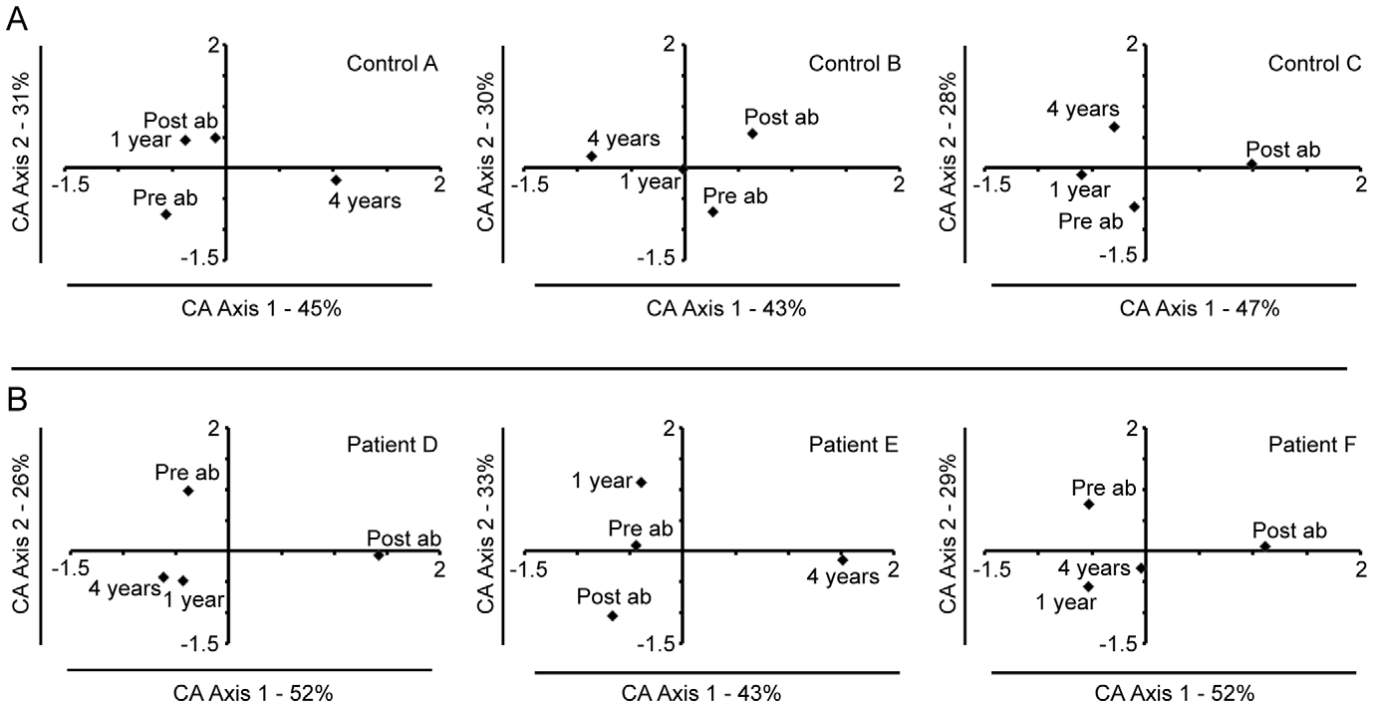
Correspondence analysis of the bacterial community found in throat samples. Each correspondence analysis plot represents the relative abundance values for the OTUs from the 16S rRNA pyrosequencing at day 0, day 8–13, 1 and 4 years. A: Controls; A, B, and C. B: Antibiotic treated patients; D, E, and F. Percentages of inter-sample variation explained, by the two axes are shown in the figures. In controls A–C and patients D–F the third axis represented 25%, 27%, 26%, 22%, 24%, and 20% of the variation.

**Figure 6:**
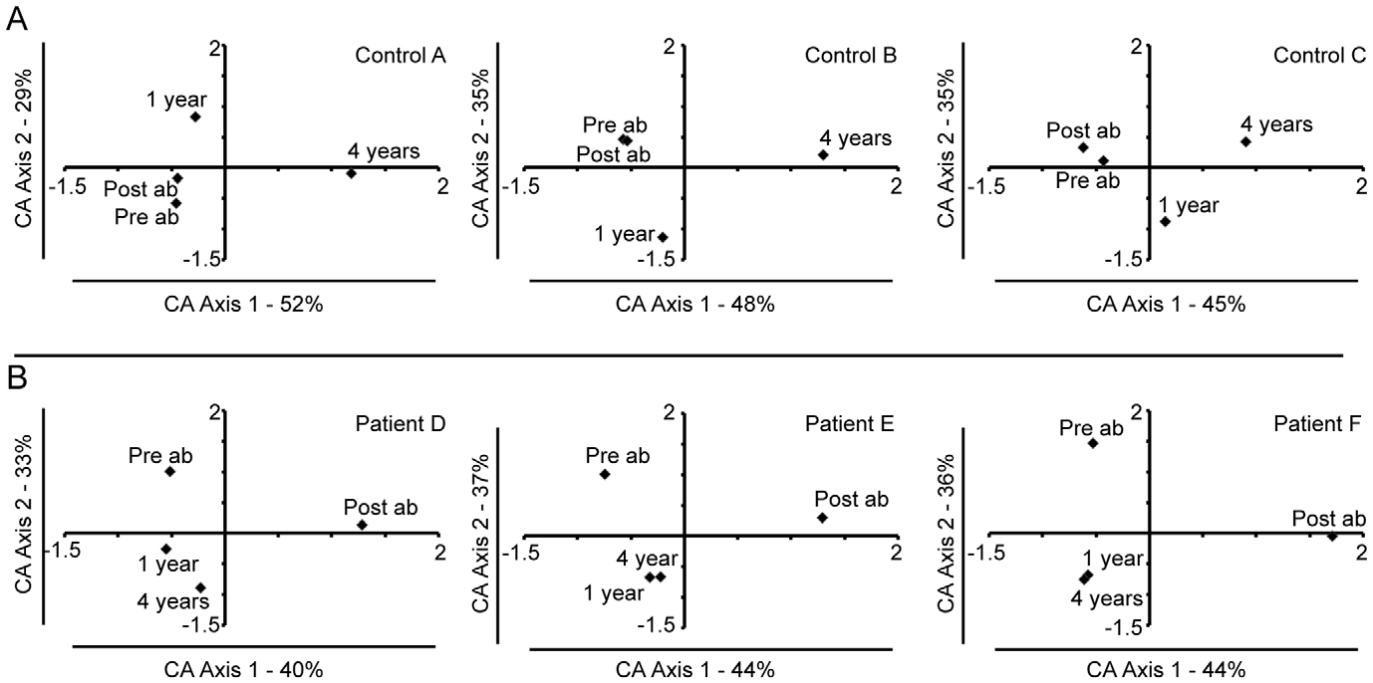
Correspondence analysis of the bacterial community found in fecal samples. Each correspondence analysis plot represents the relative abundance values for the OTUs from the 16S rRNA pyrosequencing at day 0, day 8–13, 1 and 4 years. A: Controls; A, B, and C. B: Antibiotic treated patients; D, E, and F. Percentages of inter-sample variation explained, by the two axes are shown in the figures. In controls A–C and patients D–F the third axis represented 19%, 17%, 20%, 26%, 20%, and 20% of the variation.

The microbiota of the untreated controls generally displayed lower variation within individuals than between individuals at any sampling period, as deduced by Bray-Curtis analysis of OTU frequencies (Fig. 4 and Table 1), supporting previous reports regarding a stable individual gut microbiota [1], [23], [30], [31], [32]. However, a decline in the individual gut microbiota similarity over time was observed in the control group where higher similarities were found in 5 of 6 samples collected on days 0 and 8–13 compared to day 0 and year 4 (Fig. 4, Table 1). Interestingly, the throat microbiota composition was more similar between individuals, suggesting that a more general and select microbiota is found in the human throat. These data were supported using correspondence analysis (CA), also based on OTU frequencies. In the CA analyses no time dependent clustering was found for the throat communities (Fig. 5A), however a time-dependent decline in similarity for the fecal control samples (Fig. 6A).

**Table 1:** Intra-and inter-subject Bray-Curtis values for throat and fecal samples.

Intra-subject Throat				Intra-subject Feces			
	Day 0 vs. day 8–13	Day 0 vs. 1 year	Day 0 vs. 4 years		Day 0 vs. day 8–13	Day 0 vs. 1 year	Day 0 vs. 4 years
A	0.46	0.45	0.62	A	0.39	0.52	0.65
B	0.45	0.42	0.51	B	0.37	0.65	0.74
C	0.44	0.40	0.43	C	0.36	0.46	0.50
D	0.77	0.47	0.48	D	0.80	0.66	0.71
E	0.75	0.66	0.84	E	0.80	0.67	0.69
F	0.72	0.42	0.45	F	0.99	0.85	0.86

Inter-subject Throat	Mean value ± standard deviation	Inter-subject Feces	Mean value ± standard deviation
A–B	0.53±0.07	A–B	0.68±0.06
A–C	0.59±0.11	A–C	0.82±0.04
B–C	0.58±0.10	B–C	0.86±0.05

A Bray Curtis value of 0 means the two sites has the same composition and 1 means the two sites do not share any species. Controls (A, B, C) and patients (D, E, F). For the inter-subject Bray-Curtis value, all samples and time points were compared and a mean Bray-Curtis value was calculated.

### Transient and persistent effects of antibiotic treatment

**Figure 7:**
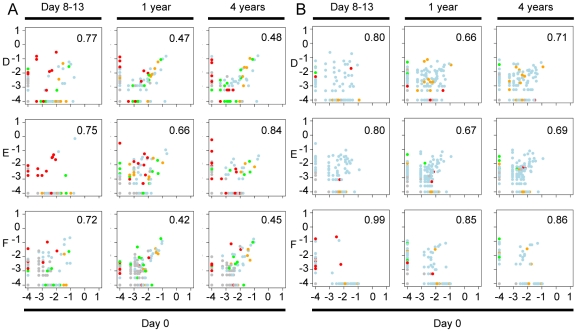
Correlation plots for the patients. Correlation plots showing OTU frequency at day 0 (x-axis), day 8–13, 1 and 4 years (y-axis) in throat (A) and fecal (B) samples in the patients (D, E, and F). Bray-Curtis values are indicated as numbers in the figure. A Bray Curtis value of 0 suggest the two sites have the same composition and 1 that the two sites do not share any species. The colors of the dots represent different phyla: yellow, Actinobacteria; green, Bacteroidetes; blue, Firmicutes; red, Proteobacteria; grey, other phyla.

In contrast to the situation for the controls, the throat and fecal microbiotas in treated patients differed considerably from their pre-treatment compositions according to Bray-Curtis analysis (Fig. 7A and 7B) and by CA analysis (Fig. 5B and 6B). The post treatment sample (immediately after treatment) was separated from the pre-treatment sample by CA axis 1, which describes the largest variation, in all fecal samples and in two out of three throat samples (Fig. 6B, 5B). One and four years after treatment, the fecal microbial communities had partially recovered to their pre-treatment compositions, however these were still separated along CA axis 2 (Fig. 6B). The decline in similarity over time was particularly evident for the fecal microbiota of patient F, and the throat community of patient E (Table 1). The bacterial diversity (Shannon diversity index) in the samples also decreased immediately following treatment (Table S5), which was also reflected in the rarefraction curves (Figs. S5 and S6). This decline in diversity was most pronounced in the fecal samples. However at the later sampling periods the bacterial diversity was more similar to that found prior to treatment (Table S5, Figs. S5 and S6).

Immediately after antibiotic treatment the average throat microbiota of the three treated individuals were dominated mainly by three bacterial phyla; Firmicutes (54%), Proteobacteria (33%), and Fusobacteria (4%), whereas their fecal microbial communities were dominated by only two phyla; Firmicutes (78%) and Proteobacteria (19%) (For individual values see Tables S2 and S3). The Actinobacteria levels were particularly reduced in the throat and gut environments of all three subjects immediately after antibiotic treatment (Figs. 1 and 2, Tables S2 and S3). This effect was most pronounced in the fecal samples where the majority of Actinobacteria belonged to the subclasses Actinobacteridae and Coriobacteridae. Very few or no reads corresponding to Bacteroidetes were detected in feces of the patients at day 0, thereby negating the possibility to draw conclusions regarding the impact of the treatment on this phylum.

### Individuality in antibiotic response

In addition to the overall trends in community change, the pyrosequencing data showed that specific members of the microbiota of each individual were impacted differently by the antibiotic treatment (Fig. 3A and B), presumably due to their unique microbiota compositions prior to treatment. In general, negatively affected taxonomic groups in throat samples were *Actinomyces* and *Prevotella* in all samples, and *Coprococcus* in two of the three patient samples (Fig. 3A), whereas negatively affected taxonomic groups in the majority of the fecal samples were *Bifidobacterium*, *Collinsella*, and *Ruminococcus* (Fig. 3B). Due to the individual responses found, each individual will be separately discussed in the following section.

Individual D. The throat sample showed a decrease in members of the Firmicutes immediately after treatment to only 19% of the total microbiota (Table S2). One genus negatively affected within the Firmicutes was *Coprococcus* (from relative abundance 18% to 0%). The dominant phylum in the throat of this patient after antibiotic treatment was instead Proteobacteria (61%), and examples of positively affected taxonomic groups within this phylum were *Neisseria* (from relative abundance 1% to 29%) and unclassified *Enterobacteriaceae* (from relative abundance 0% to 28%) (Fig. 3A). At the 1 and 4 year sampling times the throat microbiota was dominated by four bacterial phyla: Firmicutes, Proteobacteria, Actinobacteria, and Bacteriodetes. Also, the increase of the unclassified *Enterobacteriaceae* persisted in this patient during the 4-year time period (Fig. 3A).

In feces, the major effect was a negative impact on some members of the Actinobacteria, where *Bifidobacterium* and *Collinsella* were the major taxonomic groups affected (Fig. 3B). A positive impact in feces was seen for unclassified *Lachnospiraceae* that increased in relative abundance from 19% to 48%.

Individual E. After antibiotic treatment the relative levels of the Firmicutes increased in relative abundance from 53% to 81% in the throat samples of this individual (Table S2). There was also an increase in Proteobacteria post treatment that persisted and continued to increase over time (Table S2). The relative abundance of *Neisseria* also increased and persisted one year after treatment (Fig. 3A). After treatment *Haemophilus* and *Streptococcus* also increased from 1% to 9% and 28% to 71%, respectively, of the community (Fig. 3A).

In the feces the *Enterococcus* sp. within the Firmicutes phylum were positively affected immediately after treatment (Fig. 3B, Fig. S2), as also seen in the T-RFLP data, and increased from 0 to 28% relative abundance. However, no sequences corresponding to *Enterococcus* sp. were detected at the later sampling periods.

Individual F. In the throat samples the relative abundances of Actinobacteria and Bacteroidetes decreased and Fusobacteria, Proteobacteria and Firmicutes increased after antibiotic treatment (Table S2). Within the Actinobacteria and Bacteroidetes phyla, *Actinomyces* and *Prevotella* in particular decreased in relative abundance from 10% to 0% and 12% to 3%, respectively (Fig. 3A).

In the fecal samples, Proteobacteria (*Klebsiella* genus) had the highest relative abundance after treatment (Fig. 3B). Similar to individual E, the relative abundance of the *Enterococcus* sp. increased immediately after antibiotic treatment, from 0% to 11% relative abundance (Fig. 3B and Fig. S3) as also observed by T-RFLP, whereas unclassified *Lachnospiraceae* decreased in relative abundance 29% to 0%.

### Long-term persistence of antibiotic resistance genes

The antibiotic resistance gene, *erm*(B), was not detected in the throat samples of the treated individuals prior to treatment on day 0 (data not shown). Directly after antibiotic treatment the *erm*(B) gene varied around baseline levels (range 8–400 fold increase) and it persisted at this level up to 4 years. The *erm*(B) gene was also detected in the three controls analyzed at all sampling occasions, and these levels also varied around baseline levels (data not shown).

**Figure 8:**
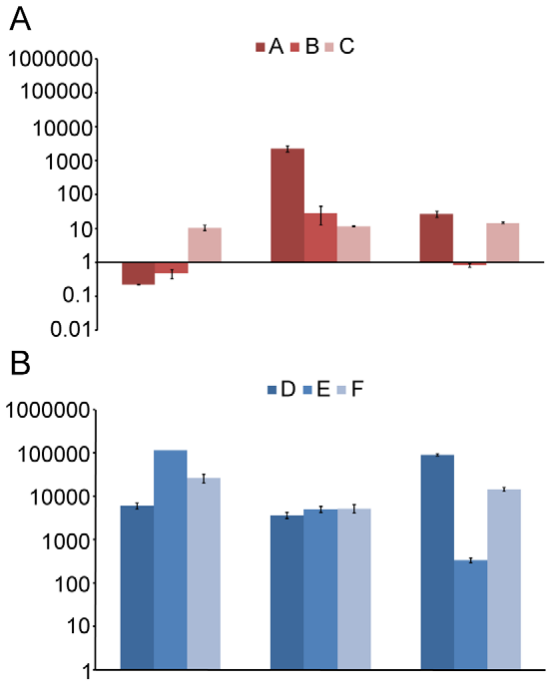
*erm*(B) abundance over time. The normalized fold increase of *erm*(B) compared to day 0 in community DNA extracted from fecal samples for controls (A–C) not receiving any treatment (A) and patients (D–F) receiving antibiotics (B). Each bar graph represents the mean and standard error of the normalized expression of *erm*(B) compared to 16S. Normalization was carried out as previously been described [8].

There was also a low abundance or no detectable amount of *erm*(B) in the fecal samples prior to treatment (data not shown). The *erm*(B) gene also remained around baseline levels in the fecal samples from the controls, except in subject A, that had a detectable increase in the 1 year sample (Fig. 8A). By contrast, the *erm*(B) gene levels increased dramatically by 3 to 5 orders of magnitude in the antibiotic treated subjects immediately after treatment (Fig. 8B). After 1 year, there was still over a 1000-fold increase in *erm*(B) levels in all three patients and the persistence was still evident 4 years post treatment in all treated subjects, but especially in patients D and F (Fig. 8B).

## Discussion

We used a combination of molecular approaches to monitor ecological disturbances of the human microbiome in individuals treated with clarithromycin and metronidazole. We found that treatment with clarithromycin and metronidazole had a long-term impact on the gut microbiota. A lower diversity of the gut microbiota was found in the throat and fecal samples immediately after treatment. However, the patient number was too few to calculate whether these observations were significant or not. For future studies it would be valuable to analyze more samples before onset of treatment for each individual to establish a better baseline.

Previous studies using cultivation-based approaches have shown that these antibiotics resulted in short-term ecological effects on the indigenous gut microbiota [7], [20], [21]. Adamsson et al. [7] found that the numbers of culturable *Bifidobacterium*, *Clostridium*, and *Bacteroides* spp. significantly decreased in feces after treatment, while the numbers of enterococci significantly increased one week after treatment. They also detected a persistent decrease of *Bifidobacterium* sp. and *Bacteroides* sp. 4 weeks after treatment. The molecular approaches that we used enabled us to explore the impact of treatment in more depth compared to cultivation. As previously described [7], we also found relative decreases in members of the genera *Clostridia* and *Bifidobacteria* and increases in the enterococci in fecal samples from treated individuals. Negligible sequences corresponding to Bacteroidetes were detected in feces collected from patients at day 0. This could be due to bias in the DNA extraction method used, although Bacteroidetes have previously been detected at abundant levels in fecal samples from other individuals using the same extraction methods [8] and amplification protocols [29] in our group. Another possibility is that Bacteroidetes is specifically under-represented in these individuals, since it has previously been shown that this phylum has strong inter-subject variability [23]. The 16S rRNA primers used for the pyrosequencing in the present study were evaluated in a previous study and showed no specific bias regarding Bacteroidetes [24].

A few recent studies have pointed to an underestimation of Actinobacteria in feces in relation to other phyla due to primer bias [24], [33], but the primers used in the present study successfully amplified Actinobacteria [24]. Increasing evidence has linked some members of the Actinobacteria to human health [34], [35]. For example, an increased abundance of Actinobacteria has recently been associated with obesity [36]. Although only a limited number of studies have been conducted to date, together they highlight the importance of studying Actinobacteria in more detail. In the present study we found that Actinobacteria were strongly reduced, especially in feces, by a commonly used antibiotic treatment.

We studied the impact of the antibiotic regimen on the microbiota in the throat in addition to the gut. The oral cavity has a large surface area with several distinct niches; each suggested to harbor a specific bacterial microbiota according to a recent study of the healthy microbiota in different body sites [37]. Our results indicate that the throat microbiota was more similar between individuals and more stable after antibiotic treatment and over long periods than the intestinal microbiota, suggesting that the throat environment is more selective for a specific microbiota. Other studies have also found that the most common genera in the throat and oral cavity are members of the *Streptococcus*, *Gemella*, *Veillonella*, *Actinomyces*, *Rothia*, *Neisseria* and *Prevotella* [24], [37]. The oral microbiota has been shown to closely resemble that found in the distal esophagus and suggested to contain a complex but conserved bacterial community [26].

The effects of treatment that were observed on the bacterial communites in the throat and feces might be due to either clarithromycin or metronidazole or both in combination. However, there are some differences in the target bacteria for the two antibiotics. The decrease of Actinobacteria is probably due to clarithromycin, which have representatives known to be susceptible to this antibiotic [38], while they are naturally resistant to metronidazole ((http://www.srga.org/). By contrast, metronidazole is known to be active against anaerobic bacteria and both antibiotics are active against *Streptococcus*. It is also important to highlight that macrolide excretion is primarily accomplished through bile, approximately 20–30% is excreted through the urine and the rest is excreted through feces. For metronidazole 10–15% is excreted into feces and the rest is excreted by urine (http://www.srga.org/).

The antibiotic treatment impacted the indigenous microbiota differently in the individual treated subjects, probably due to the known unique bacterial community compositions in different individuals [1], [23], [30], [31], [32]. We found that the microbial communities were more similar at both sampling locations within individuals than between individuals, at least up to the one year sampling period. This unique individual community composition was particularly evident for the fecal samples where larger differences were observed between individuals. There was also a pronounced temporal change in fecal community structure and after four years the gut communities had diverged in both the control group and the treated group.

Recent studies have shown that some antibiotics have long-term impacts on the fecal microbiota [8], [9], [10], [11], [17], [39], [40]. For example, clindamycin treatment resulted in a lower *Bacteroides* diversity in fecal samples, an enrichment of resistant *Bacteroides* clones, specifically *B. thetaiotamicron*, and an increase in resistance *erm*-genes up to two years after treatment [8]. Long-term impacts (six months) were also recently seen in the gut microbiota after treatment with ciprofloxacin [39]. Although the majority of the gut microbiota returned to pretreatment levels after four weeks, some taxa failed to recover to pretreatment levels for periods up to 6 months.

Another clear indication of a persistent impact of the antibiotic treatment on the gut microbiota was a marked increase of *erm*(B) gene levels in the fecal samples that persisted up to four years after treatment. The increase and persistence of *erm*(B) can either be explained by clonal expansion of stable, resistant isolates originally present in the intestinal microbiota pretreatment, or by *erm*(B) acquisition by new populations via horizontal gene transfer. Epidemic spread of *erm*(B) has been shown to be the cause of increased macrolide resistance in *Streptococcus pyogenes* [41]. Our results suggest a link between *erm*(B) gene levels and the increase in enterococci after treatment, indicating that the enterococci might be the bearers of antibiotic resistance. Highly macrolide-resistant enterococci have previously been reported to be selected by treatment with clarithromycin and metronidazole as evidenced by the increase in *erm*(B) levels in cultured enterococci after treatment [10]. One resistant clone persisted for three years without any further antibiotic pressure [10].

This is one of the first studies to our knowledge to thoroughly follow the dynamics of the throat microbiota over time in healthy individuals and following antibiotic treatment. Recently it was reported that clarithromycin treatment selects for macrolide-resistant bacteria in the throat microbiota [17], supporting the findings we present here. Interestingly, we found that *erm*(B) was detected in throat samples from both patients and controls in the present study, although not in a high abundance in the controls. The detection of low levels of *erm*(B) in the control samples could be explained by previous exposure to macrolides before inclusion in the study.

In conclusion, a common one-week antimicrobial treatment regimen with clarithromycin and metronidazole resulted in marked ecological disturbances in the throat and gut microbiota with potential long-term consequences. The molecular approaches that we used enabled a more detailed monitoring of ecological disturbances due to antibiotic treatment that has previously been possible using conventional cultivation techniques. In total, the observations in the present study underline the importance of restrictive and proper use of antibiotics in order to prevent long-term ecological disturbances of the indigenous microbiota. In hospital environments, a decreased colonization resistance and persistence of antibiotic resistance could potentially lead to an overgrowth and spread of multi-resistant potential pathogenic bacteria and thereby increase the risk of treatment failure.

## Materials and Methods

### Ethics statement

Written consent was obtained from all participants involved in the study and the study was approved by written consent by the human ethics committee at Uppsala University, Uppsala, Sweden.

### Subjects and treatment

Six individuals were included in this study: three control patients (A, B, and C), hereafter referred to as controls, with diagnosed dyspeptic disorders who did not receive any treatment and three patients (D, E, and F) that had either a gastric or duodenal ulcer and were treated twice a day for seven days with metronidazole 400 mg, clarithromycin 250 mg, and omeprazole 20 mg (Supplementary Table S6 for subject info). None of the patients had received any antibiotic treatment within four weeks prior to the start of the study and during the four-year course of the study no other antimicrobial treatment was allowed. The patients did not receive any diet restrictions or recommendations before inclusion into the study. A throat swab and a fecal sample were collected from each subject one day before treatment, immediately after (Control A: 13 days, control B: 12 days, control C: 13 days, patient D: 12 days, patient E: 8 days, patient F: day 10) and one and four years after treatment. All samples were stored at −70°C until analyzed. This material was part of a larger longitudinal cohort study where the aim was to study eradication of *H. pylori* using a common treatment regimen [11].

### DNA extraction

DNA was extracted from 250–500 µl throat samples from each subject. The throat samples were diluted (1∶1) in a 20 mM Tris-HCI (pH 8.0) and 2 mM sodium EDTA buffer and spun for 10 min at 5000×g. The samples were lysed in 180 µl lysozyme buffer (20 mM Tris-HCI, pH 8.0, 2 mM sodium EDTA, 1.2% Triton X-100, and 20 mg/ml lysozyme (Sigma-Aldrich, Schnelldorf, Germany) and incubated at 37°C for 1 h. Proteinase K and 200 µl Buffer AL from Qiagen's DNeasy Tissue Kit (Qiagen, Hilden, Germany) were added and the mixture was incubated at 56°C for 16 h followed by DNA extraction using Qiagen's DNeasy Tissue Kit (Qiagen), according to the manufacturer's instructions. DNA was extracted from duplicate 100 mg fecal samples from each subject using a FastDNA SPIN Kit for Soil (BIO 101, Carlsbad, Calif.) according to the manufacturer's instructions. The bead-beating step was performed in a FastPrep instrument (BIO 101) for 2 times 20 s at speed 5.5.

### 16S rRNA tag pyrosequencing

The 16S rRNA genes were amplified in a 50 µl reaction containing 1X PCR-buffer, 200 µM dNTP PurePeak DNA polymerase Mix (Pierce Nucleic Acid Technologies, Milwaukee, WI, USA), 500 µM of each primer (SGS, Köping, Sweden) and 2.5 U of *PfuUltra* High-Fidelity DNA polymerase (Stratagene La Jolla, CA, USA). The primer pairs used, targeting the V6 region of the 16S rRNA gene, were 784f with adaptor B and 1061r with adaptor A and sample-specific sequence barcodes (for primer, adaptor, and barcode sequences, see Supplementary Table S7) [24]. To each reaction 1 µl of the extracted template DNA was added. The PCR conditions used were 95°C for 5 min, 25 cycles of 95°C for 40 sec, 55°C for 40 sec and 72°C for 1 min followed by 72°C for 7 min. Three amplification reactions were performed for each sample and then pooled together. The PCR-products with a proximal length of 300 bp were excised from a 1% agarose gel stained with ethidium bromide and purified using a QIAquick gel extraction kit (Qiagen). The DNA concentration and quality were assessed on a Bioanalyzer 2100 (Agilent, Palo Alto, CA, USA) using a DNA1000 lab chip (Agilent). Equal DNA amounts of four samples with different sample specific barcode sequences were pooled to a final concentration of 10 ng/µl. The pooled DNA were amplified in PCR-mixture-in-oil emulsions and sequenced on different lanes of a 16-lane PicoTiterPlate on a Genome Sequencer FLX system (Roche, Basel, Switzerland) at the Swedish Institute for Infectious Disease Control (Solna, Sweden). In additon to the standard sequence quality filtering applied by the instrument software, pyrosequencing reads were removed that 1) did not include a correct primer sequence, 2) were shorter than 200 bp (excluding the primer sequence) and 3) included any ambiguous bases. The resulting reads were finally trimmed to 200 bp (excluding primer).

### Taxonomic classification

The 200 bp trimmed pyrosequencing reads were aligned and clustered into operational taxonomic units (OTUs) with complete linkage clustering at maximum within-cluster distance of 3% using the RDP pyrosequencing pipeline (http://rdp.cme.msu.edu/). The most abundant 454 sequence (one per group of identical sequences) within one OTU were BLAST searched with default parameters against a local database comprising 269,420 near full-length bacterial 16S rRNA gene sequences from the Ribosomal Database Project (RDP) v. 10.7 [42]. The sequences inherited the taxonomic annotation (down to genus level) of the best scoring RDP hit fulfilling the criteria of ≥95% identity over an alignment of length ≥180 bp. If no such hit was found the sequence was classified as “no match”. Details regarding primer design is found in a previous publication [24]. Rarefraction analysis on the throat and fecal microbiota was performed using Analytical Rarefraction 1.3 (http://www.uga.edu/strata/software/).

### T-RFLP

16S rRNA genes were amplified in the DNA extracts using the eubacterial primers fD1-FAM [43] labeled at the 5′ end with 6-carboxyflourescein and 926r [44] (for primer sequences, see Supplementary Table S8). All primers were synthesized by Invitrogen (Carlsbad, Calif.) Amplification was carried out in a 50 µl reaction mixture containing 2.5 U of *Taq* DNA polymerase (Amersham Biosciences, Uppsala, Sweden), 1X PCR Buffer (Amersham Biosciences), 0.7 µmol of each primer, 10 nmol of each deoxynucleoside triphosphate (Amersham Pharmacia Biotech), 2.5 µl dimethyl sulfoxide, sterile distilled water, and 1 µl of template DNA. The cycling program was performed in a Perkin-Elmer GeneAmp PCR system 2400 thermocycler using the following conditions: 95°C for 5 min followed by 25 cycles of 95°C for 40 s, 55°C for 40 s, and 72°C for 1 min. The last cycle ended with an elongation step for 7 min. The restriction enzyme digestions, electrophoresis conditions and calculations were performed as previously described [29].

### Real-time PCR

The abundance of *erm*(B) genes was analyzed in the throat and fecal samples. The 16S rRNA gene was used as a reference gene. Primers and probes were designed using Primer Express software 2.0 (ABI). The *erm*(B) gene was amplified using *erm*Bf/*erm*Br and a *Taq*Man probe (ABI). The 16S rRNA genes were amplified using 16Sf/16Sr and a *Taq*Man probe. The fluorescent reporter dye at the 5′ end of the probe was 6-FAM; the quencher at the 3′ end was a black-hole quencher-1 (BHQ-1). For primer and probe sequences, see Supplementary Table S8. All primers were synthesized using Invitrogen (Carlsbad, Calif.) and the probes were synthesized using Thermo Electron GmbH (Ulm, Germany). The cycling program was performed on an ABI Prism 7900HT (ABI) under the following conditions: 2 min at 50°C, 10 min at 95°C, followed by 40 cycles of 15 s at 94°C and 1 min at 60°C. The PCR mixture without template DNA was included in each run as a negative control. The results were analyzed using the software SDS 2.1 (ABI). In the present study we normalized the *erm*(B) gene copies to the number of 16S copies and preparation of curves and calculations were carried out as previously described [8].

### Statistical analysis and diversity estimations

Shannon diversity index was calculated as −Σ log(p*_i_*)p*_i_* [45], where p*_i_* denotes the frequency of phylotype. Significance was tested using Wilcoxon t-test in the R software (http://www.r-project.org/). Bray-Curtis similarity was also calculated using the R software. Correspondence analysis (CA) was performed on the pyrosequencing data using the General Rweb interface (http://pbil.univ-lyon1.fr/Rweb/). The relative abundance values of each OTU were used in the calculation and scatter plots were created. The 454 sequences from the samples were clustered in TMEV (http://www.tm4.org/mev.html).

## Supporting Information

Figure S1 T-RFLP peaks from patient D fecal sample. Relative abundance values of TFRs obtained using the general eubacterial 16S rRNA primers fD1 and 926r and *Hae*III restriction digestion for patient D. The 273-bp TRF represents members of *Clostridium coccoides* subgroup such as *Clostridium clostridiiforme* ATCC 25537 and *Eubacterium formicigenerans* ATCC 27755 and the 275-bp TRF represents *Eubacterium ramulus* ATCC 29099, *Eubacterium rectale* ATCC 33656, *Eubacterium ventriosum* ATCC 27560, and *Roseburia cecicola* ATCC 33874.
(0.81 MB TIF)

Figure S2 T-RFLP peaks from patient E fecal sample. Relative abundance values of TFRs obtained using the general eubacterial 16S rRNA primers fD1 and 926r and *Hae*III restriction digestion in patient E. The 273-bp TRF represents members of *Clostridium coccoides* subgroup such as *Clostridium clostridiiforme* ATCC 25537 and *Eubacterium formicigenerans* ATCC 27755 and the 275-bp TRF represents *Eubacterium ramulus* ATCC 29099, *Eubacterium rectale* ATCC 33656, *Eubacterium ventriosum* ATCC 27560, and *Roseburia cecicola* ATCC 33874. The 285-bp TRF represents *Enterococcus faecalis*.
(0.34 MB TIF)

Figure S3 T-RFLP peaks from patient F fecal sample. Relative abundance values of TFRs obtained using the general eubacterial 16S rRNA primers fD1 and 926r and *Hae*III restriction digestion in patient F. The 273-bp TRF represents members of *Clostridium coccoides* subgroup such as *Clostridium clostridiiforme* ATCC 25537 and *Eubacterium formicigenerans* ATCC 27755 and the 275-bp TRF represents *Eubacterium ramulus* ATCC 29099, *Eubacterium rectale* ATCC 33656, *Eubacterium ventriosum* ATCC 27560, and *Roseburia cecicola* ATCC 33874. The 285-bp TRF represents *Enterococcus faecalis*. The 242-bp TRF was unidentified.
(0.36 MB TIF)

Figure S4 Taxonomic groups found in throat or fecal samples. The heat maps show the relative abundance per sample of different taxonomic groups found in throat or fecal control samples. The color panel shows the percent relative abundance (0–70%) of different taxonomic groups within the major phyla (Actinobacteria, Bacteroidetes, Firmicutes, Fusobacterium and Proteobacteria) detected in throat (A) and fecal (B) samples from controls at time points: day 0 (1), day 8–13 (2), 1 year (3) and 4 years (4).
(0.20 MB TIF)

Figure S5 Rarefraction analysis of throat samples. Number of phylotypes sampled as a function of number of reads in controls (A–C) and patients (D–F). Sampling time points: A = Day 0, B = Day 8–13, C = 1 year, D = 4 years.
(0.45 MB TIF)

Figure S6 Rarefraction analysis of fecal samples. Number of phylotypes sampled as a function of number of reads in controls (A–C) and patients (D–F). Sampling time points: A = Day 0, B = Day 8–13, C = 1 year, D = 4 years.
(0.44 MB TIF)

Table S1 Number of reads, OTUs and genus per individual (A–F).
(0.04 MB DOC)

Table S2 Individual relative abundance values (%) for the dominant phyla found in throat samples over time. The sequences inherited the taxonomic annotation (down to genus level) and the best scoring RDP hit fulfilling the criteria of ≥95% identity over an alignment of length ≥180 bp. If no such his was found the sequence was classified as “no match”.
(0.05 MB DOC)

Table S3 Individual relative abundance values (%) for dominant phyla found in fecal samples over time. The sequences inherited the taxonomic annotation (down to genus level) and the best scoring RDP hit fulfilling the criteria of ≥95% identity over an alignment of length ≥180 bp. If no such his was found the sequence was classified as “no match”.
(0.04 MB DOC)

Table S4 Dominant taxonomic groups found in throat- and fecal samples. The relative abundance (in percentage) of the 20 most dominant taxonomic groups found throat and fecal samples, respectively. The abundance of all OTUs belonging to the same genus, or were unclassified at the same taxonomic level, were summed.
(0.04 MB DOC)

Table S5 Diversity estimations. Shannon diversity index was calculated based on OTU frequency. The diversity index decreased at day 8–13 in both throat- and fecal samples following antibiotic treatment while it remained stable within the controls during the whole time period.
(0.04 MB DOC)

Table S6 Features of participants included in the study.
(0.03 MB DOC)

Table S7 Oligonucleotides, adaptor, and sample specific barcode sequences for 16S rRNA sequencing.
(0.03 MB DOC)

Table S8 Oligonucleotides for terminal-restriction fragment length polymorphism and real-time PCR.
(0.03 MB DOC)
