# Supplementary figures and images for: Short-Term Antibiotic Treatment Has Differing Long-Term Impacts on the Human Throat and Gut Microbiome
Source: PLoS One. 2010 Mar 24;5(3):e9836. doi: 10.1371/journal.pone.0009836 (PMC2844414; doi:10.1371/journal.pone.0009836)

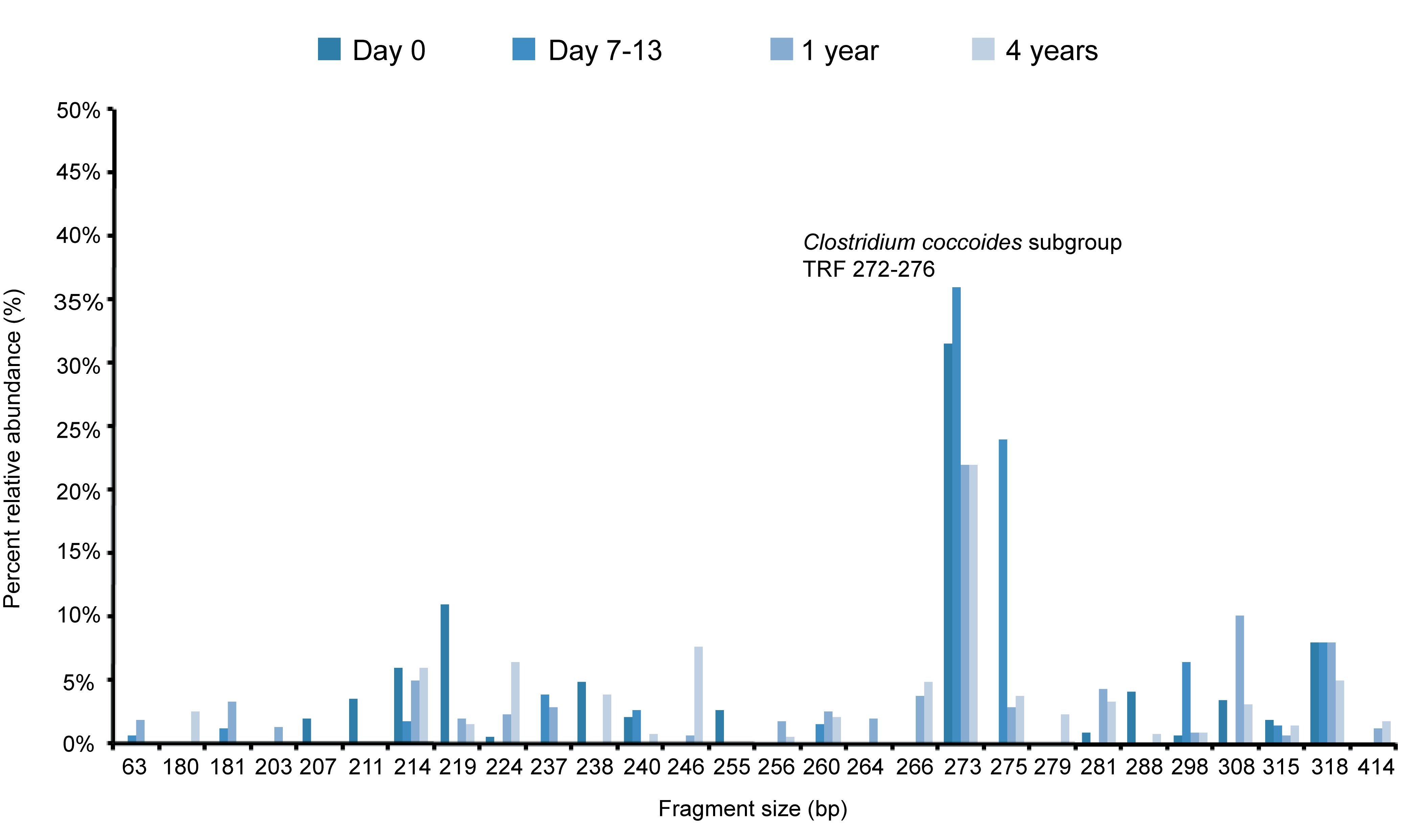

Supplement: Figure S1 — T-RFLP peaks from patient D fecal sample. Relative abundance values of TFRs obtained using the general eubacterial 16S rRNA primers fD1 and 926r and HaeIII restriction digestion for patient D. The 273-bp TRF represents members of Clostridium coccoides subgroup such as Clostridium clostridiiforme ATCC 25537 and Eubacterium formicigenerans ATCC 27755 and the 275-bp TRF represents Eubacterium ramulus ATCC 29099, Eubacterium rectale ATCC 33656, Eubacterium ventriosum ATCC 27560, and Roseburia cecicola ATCC 33874. (0.81 MB TIF) [file pone.0009836.s001.tif]

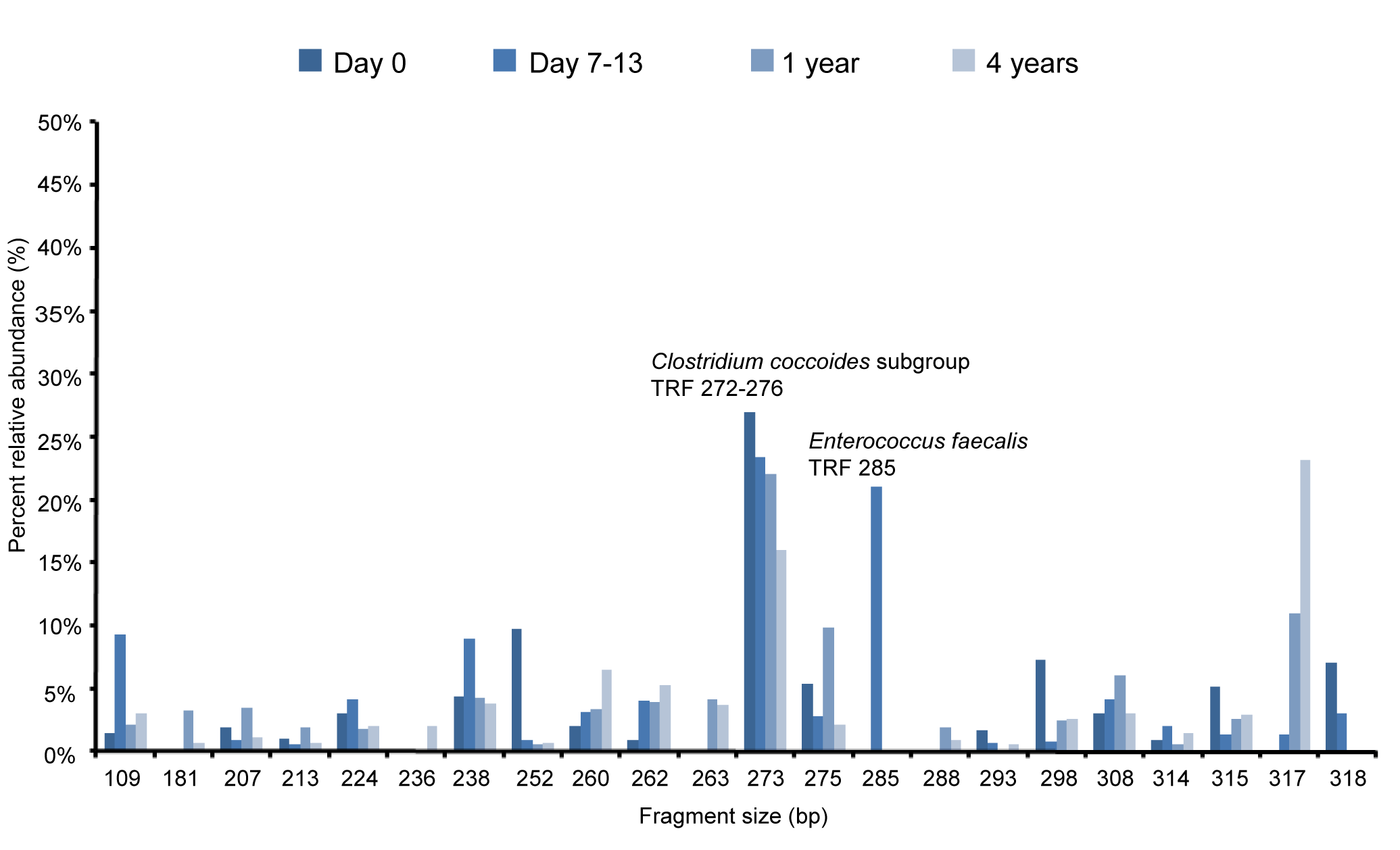

Supplement: Figure S2 — T-RFLP peaks from patient E fecal sample. Relative abundance values of TFRs obtained using the general eubacterial 16S rRNA primers fD1 and 926r and HaeIII restriction digestion in patient E. The 273-bp TRF represents members of Clostridium coccoides subgroup such as Clostridium clostridiiforme ATCC 25537 and Eubacterium formicigenerans ATCC 27755 and the 275-bp TRF represents Eubacterium ramulus ATCC 29099, Eubacterium rectale ATCC 33656, Eubacterium ventriosum ATCC 27560, and Roseburia cecicola ATCC 33874. The 285-bp TRF represents Enterococcus faecalis. (0.34 MB TIF) [file pone.0009836.s002.tif]

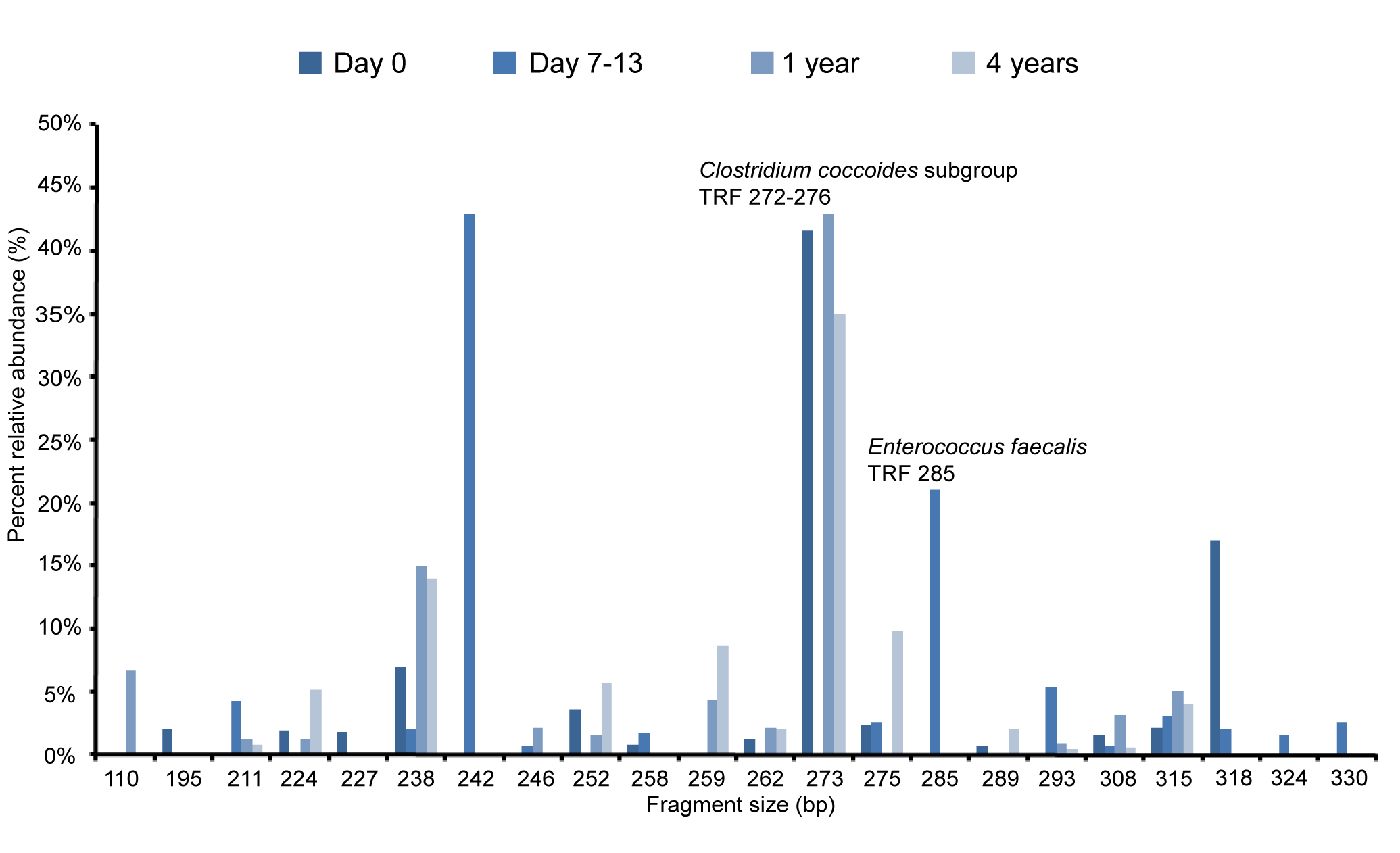

Supplement: Figure S3 — T-RFLP peaks from patient F fecal sample. Relative abundance values of TFRs obtained using the general eubacterial 16S rRNA primers fD1 and 926r and HaeIII restriction digestion in patient F. The 273-bp TRF represents members of Clostridium coccoides subgroup such as Clostridium clostridiiforme ATCC 25537 and Eubacterium formicigenerans ATCC 27755 and the 275-bp TRF represents Eubacterium ramulus ATCC 29099, Eubacterium rectale ATCC 33656, Eubacterium ventriosum ATCC 27560, and Roseburia cecicola ATCC 33874. The 285-bp TRF represents Enterococcus faecalis. The 242-bp TRF was unidentified. (0.36 MB TIF) [file pone.0009836.s003.tif]

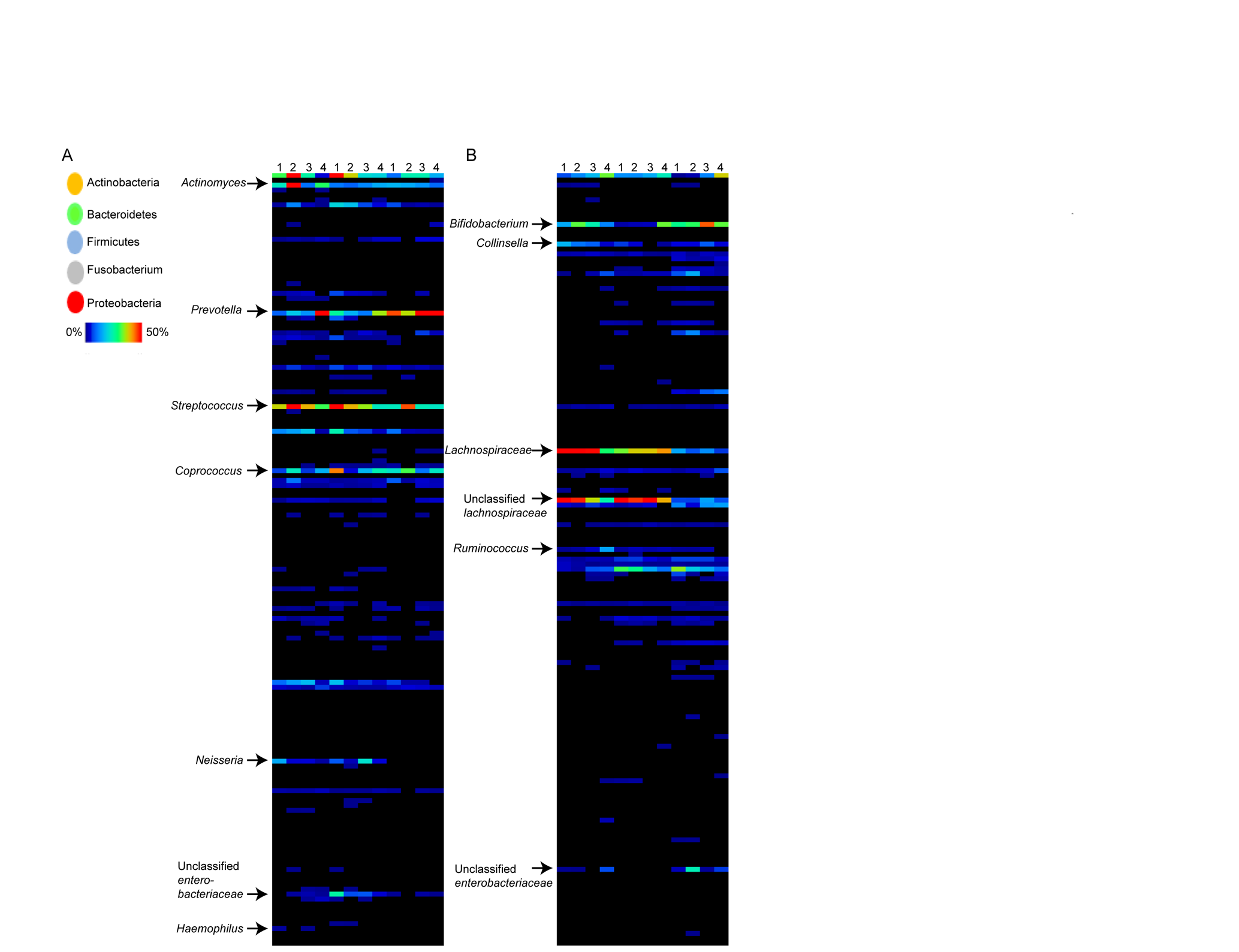

Supplement: Figure S4 — Taxonomic groups found in throat or fecal samples. The heat maps show the relative abundance per sample of different taxonomic groups found in throat or fecal control samples. The color panel shows the percent relative abundance (0–70%) of different taxonomic groups within the major phyla (Actinobacteria, Bacteroidetes, Firmicutes, Fusobacterium and Proteobacteria) detected in throat (A) and fecal (B) samples from controls at time points: day 0 (1), day 8–13 (2), 1 year (3) and 4 years (4). (0.20 MB TIF) [file pone.0009836.s004.tif]

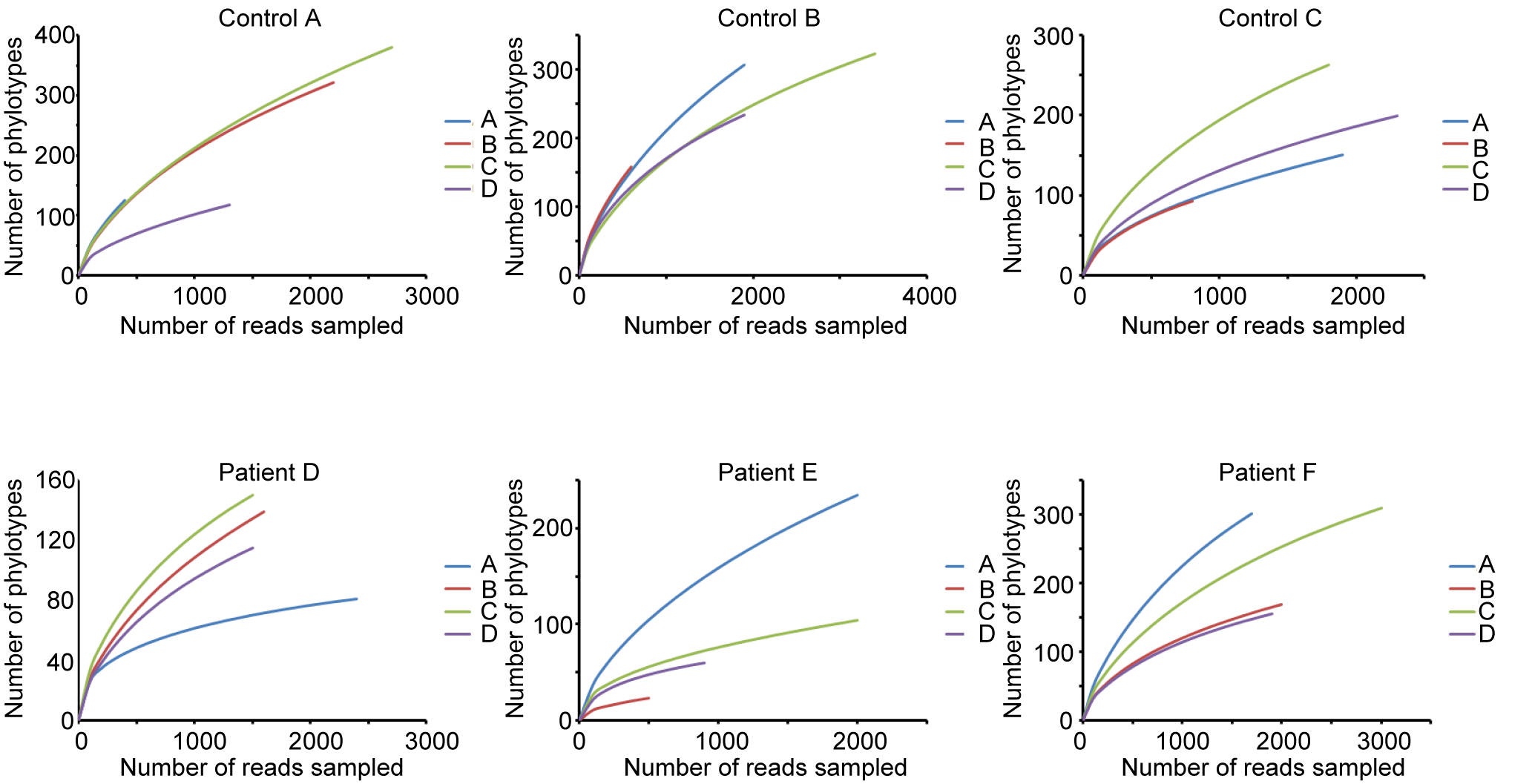

Supplement: Figure S5 — Rarefraction analysis of throat samples. Number of phylotypes sampled as a function of number of reads in controls (A–C) and patients (D–F). Sampling time points: A = Day 0, B = Day 8–13, C = 1 year, D = 4 years. (0.45 MB TIF) [file pone.0009836.s005.tif]

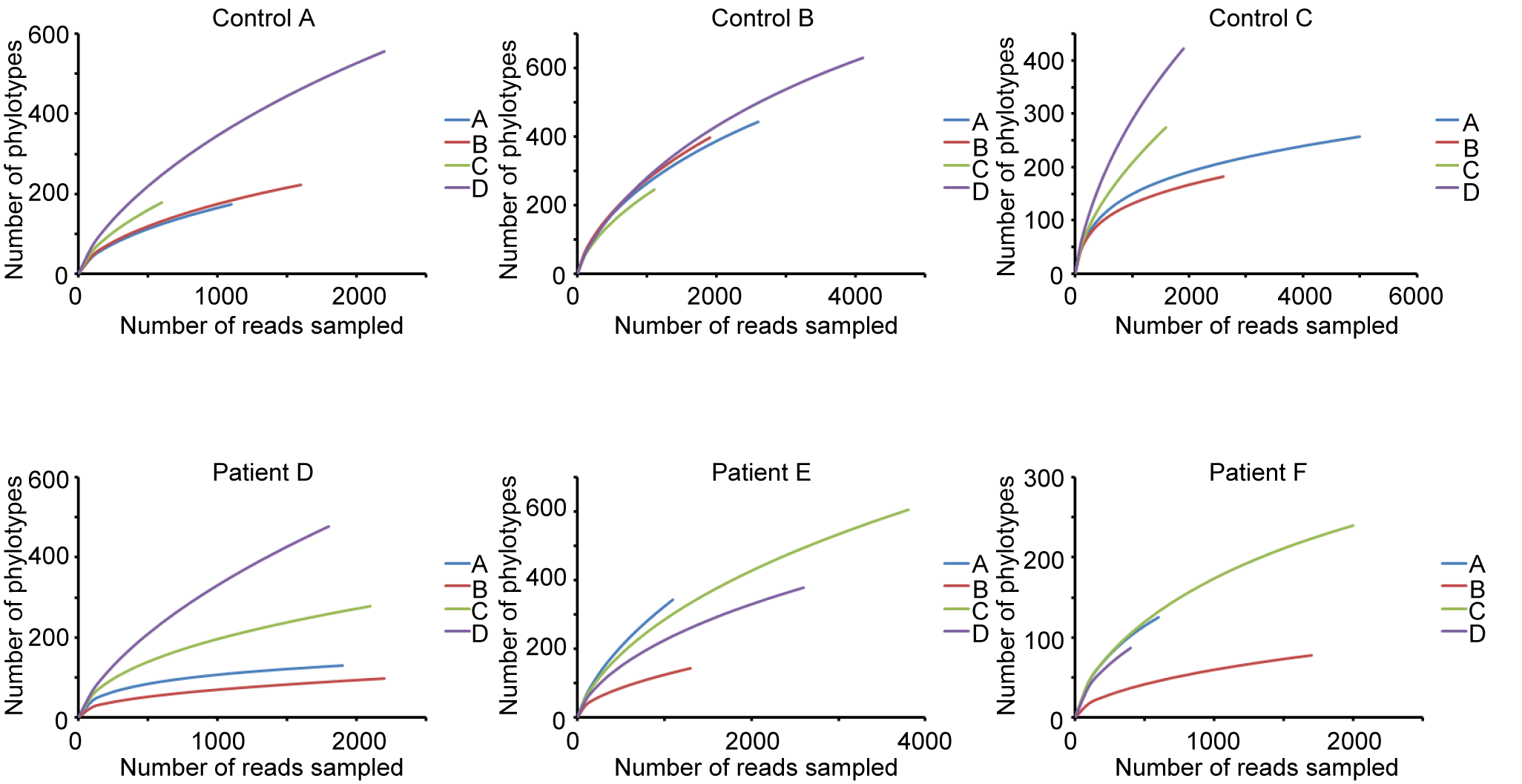

Supplement: Figure S6 — Rarefraction analysis of fecal samples. Number of phylotypes sampled as a function of number of reads in controls (A–C) and patients (D–F). Sampling time points: A = Day 0, B = Day 8–13, C = 1 year, D = 4 years. (0.44 MB TIF) [file pone.0009836.s006.tif]
