# Supplementary material for: Short-Term Antibiotic Treatment Has Differing Long-Term Impacts on the Human Throat and Gut Microbiome
Source: PLoS One. 2010 Mar 24;5(3):e9836. doi: 10.1371/journal.pone.0009836 (PMC2844414; doi:10.1371/journal.pone.0009836)
Supplement: Table S1 — Number of reads, OTUs and genus per individual (A–F). (0.04 MB DOC) [file pone.0009836.s007.doc]

Table S1. Number of reads, OTUs and genus per individual (A-F).

| Sequence | Individual | Total no of  reads | No of OTUs | No of OTUs taxonomically  classified to a genus |
| --- | --- | --- | --- | --- |
| V6 region throat | A | 9218 | 975 | 374 |
|  | B | 10160 | 1046 | 370 |
|  | C | 7209 | 669 | 289 |
|  | D | 7277 | 493 | 261 |
|  | E | 7728 | 428 | 228 |
|  | F | 10896 | 944 | 338 |
| V6 region feces | A | 5764 | 1205 | 508 |
|  | B | 9984 | 1737 | 717 |
|  | C | 11401 | 1147 | 475 |
|  | D | 8177 | 958 | 474 |
|  | E | 9094 | 1494 | 662 |
|  | F | 4921 | 538 | 284 |
