# Supplementary material for: Short-Term Antibiotic Treatment Has Differing Long-Term Impacts on the Human Throat and Gut Microbiome
Source: PLoS One. 2010 Mar 24;5(3):e9836. doi: 10.1371/journal.pone.0009836 (PMC2844414; doi:10.1371/journal.pone.0009836)
Supplement: Table S2 — Individual relative abundance values (%) for the dominant phyla found in throat samples over time. The sequences inherited the taxonomic annotation (down to genus level) and the best scoring RDP hit fulfilling the criteria of ≥95% identity over an alignment of length ≥180 bp. If no such his was found the sequence was classified as “no match”. (0.05 MB DOC) [file pone.0009836.s008.doc]

| Throat | | | | | | | | | | | | | | |
| --- | --- | --- | --- | --- | --- | --- | --- | --- | --- | --- | --- | --- | --- | --- |
|  | Control A | | | | Control B | | | | | | Control C | | | |
| Phylum | Day 0 | Day 7-13 | 1 year | 4 years | Day 0 | Day 7-13 | | 1 year | | 4 years | Day 0 | Day 7-13 | 1 year | 4 years |
| Actinobacteria | 15% | 24% | 8% | 20% | 9% | 16% | | 12% | | 15% | 16% | 10% | 12% | 11% |
| Bacteroidetes | 8% | 8% | 14% | 31% | 14% | 13% | | 11% | | 23% | 28% | 22% | 36% | 43% |
| Proteobacteria | 10% | 2% | 6% | 5% | 11% | 7% | | 19% | | 7% | 1% | 0% | 1% | 1% |
| Firmicutes | 40% | 44% | 46% | 36% | 41% | 37% | | 42% | | 41% | 43% | 50% | 33% | 35% |
| Fusobacteria | 8% | 17% | 11% | 5% | 19% | 4% | | 4% | | 3% | 6% | 4% | 3% | 2% |
| No match | 19% | 5% | 15% | 3% | 6% | 23% | | 12% | | 12% | 6% | 14% | 14% | 9% |
|  | Patient D | | | | Patient E | | | | | | Patient F | | | |
| Phylum | Day 0 | Day 7-13 | 1 year | 4 years | Day 0 | Day 7-13 | 1 year | | 4 years | | Day 0 | Day 7-13 | 1 year | 4 years |
| Actinobacteria | 14% | 6% | 12% | 17% | 17% | 0% | 7% | | 2% | | 14% | 2% | 9% | 6% |
| Bacteroidetes | 11% | 5% | 11% | 13% | 15% | 0% | 6% | | 3% | | 13% | 3% | 28% | 13% |
| Proteobacteria | 3% | 61% | 29% | 16% | 4% | 18% | 48% | | 72% | | 2% | 20% | 3% | 13% |
| Firmicutes | 70% | 19% | 40% | 50% | 53% | 81% | 37% | | 15% | | 49% | 61% | 41% | 54% |
| Fusobacteria | 2% | 0% | 0% | 1% | 2% | 1% | 1% | | 0% | | 1% | 12% | 3% | 7% |
| No match | 0% | 9% | 9% | 3% | 9% | 1% | 1% | | 8% | | 20% | 3% | 16% | 7% |

Table S2. Individual relative abundance values (%) for the dominant phyla found in throat samples over time.

Percent relative abundance (%) of dominant phyla found in throat samples in controls (A, B, and C) and patients (D, E, and F) at day 0, day 7-13, 1, and 4 years.

The sequences inherited the taxonomic annotation (down to genus level) and the best scoring RDP hit fulfilling the criteria of ≥ 95% identity over an alignment of length ≥ 180 bp. If no such his was found the sequence was classified as “no match”.
