# Supplementary material for: Short-Term Antibiotic Treatment Has Differing Long-Term Impacts on the Human Throat and Gut Microbiome
Source: PLoS One. 2010 Mar 24;5(3):e9836. doi: 10.1371/journal.pone.0009836 (PMC2844414; doi:10.1371/journal.pone.0009836)
Supplement: Table S3 — Individual relative abundance values (%) for dominant phyla found in fecal samples over time. The sequences inherited the taxonomic annotation (down to genus level) and the best scoring RDP hit fulfilling the criteria of ≥95% identity over an alignment of length ≥180 bp. If no such his was found the sequence was classified as “no match”. (0.04 MB DOC) [file pone.0009836.s009.doc]

| Feces | | | | | | | | | | | | |
| --- | --- | --- | --- | --- | --- | --- | --- | --- | --- | --- | --- | --- |
|  | Control A | | | | Control B | | | | Control C | | | |
| Phylum | Day 0 | Day 7-13 | 1 year | 4 years | Day 0 | Day 7-13 | 1 year | 4 years | Day 0 | Day 7-13 | 1 year | 4 years |
| Actinobacteria | 20% | 27% | 23% | 11% | 5% | 6% | 2% | 21% | 26% | 24% | 36% | 27% |
| Bacteroidetes | 0% | 0% | 1% | 6% | 3% | 2% | 1% | 2% | 11% | 20% | 4% | 1% |
| Proteobacteria | 0% | 0% | 0% | 6% | 0% | 1% | 0% | 0% | 2% | 15% | 0% | 4% |
| Firmicutes | 75% | 65% | 66% | 58% | 84% | 84% | 88% | 63% | 61% | 39% | 52% | 45% |
| No match | 4% | 7% | 10% | 20% | 8% | 7% | 9% | 14% | 0% | 1% | 7% | 23% |
|  | Patient D | | | | Patient E | | | | Patient F | | | |
| Phylum | Day 0 | Day 7-13 | 1 year | 4 years | Day 0 | Day 7-13 | 1 year | 4 years | Day 0 | Day 7-13 | 1 year | 4 years |
| Actinobacteria | 15% | 0% | 18% | 8% | 4% | 0% | 1% | 2% | 14% | 0% | 6% | 5% |
| Bacteroidetes | 0% | 3% | 10% | 4% | 0% | 0% | 6% | 7% | 1% | 0% | 8% | 16% |
| Proteobacteria | 4% | 2% | 1% | 0% | 2% | 0% | 0% | 1% | 1% | 55% | 1% | 0% |
| Firmicutes | 81% | 94% | 65% | 71% | 84% | 96% | 83% | 83% | 81% | 43% | 79% | 72% |
| No match | 1% | 0% | 6% | 17% | 10% | 4% | 9% | 7% | 4% | 2% | 6% | 7% |

Table S3. Individual relative abundance values (%) for dominant phyla found in fecal samples over time.

The sequences inherited the taxonomic annotation (down to genus level) and the best scoring RDP hit fulfilling the criteria of ≥ 95% identity over an alignment of length ≥ 180 bp. If no such his was found the sequence was classified as “no match”.
