# Supplementary material for: Short-Term Antibiotic Treatment Has Differing Long-Term Impacts on the Human Throat and Gut Microbiome
Source: PLoS One. 2010 Mar 24;5(3):e9836. doi: 10.1371/journal.pone.0009836 (PMC2844414; doi:10.1371/journal.pone.0009836)
Supplement: Table S4 — Dominant taxonomic groups found in throat- and fecal samples. The relative abundance (in percentage) of the 20 most dominant taxonomic groups found throat and fecal samples, respectively. The abundance of all OTUs belonging to the same genus, or were unclassified at the same taxonomic level, were summed. (0.04 MB DOC) [file pone.0009836.s010.doc]

Table S4. Dominant taxonomic groups found in throat- and fecal samples.

| Taxonomic groups in throat | (%) | Taxonomic groups in feces | (%) |
| --- | --- | --- | --- |
| Streptococcus | 23 | Lachnospiraceae Incertae Sedis | 22 |
| Prevotella | 18 | unclassified Lachnospiraceae | 21 |
| Coprococcus | 12 | Bifidobacterium | 11 |
| Actinomyces | 11 | unclassified Ruminococcaceae | 8 |
| Gemella | 5 | Collinsella | 4 |
| Rothia | 4 | Peptostreptococcaceae  Incertae Sedis | 3 |
| Fusobacterium | 4 | Ruminococcus | 3 |
| Neisseria | 3 | unclassified Enterobacteriaceae | 2 |
| Haemophilus | 2 | Bacteroides | 2 |
| Granulicatella | 2 | Coprococcus | 2 |
| Moryella | 2 | Faecalibacterium | 1 |
| Leptotrichia | 1 | Eggerthella | 1 |
| Atopobium | 1 | Lactobacillus | 1 |
| Oribacterium | 1 | unclassified Clostridiales | 1 |
| unclassified Lachnospiraceae | 1 | unclassified Bacteroidales | 1 |
| Peptostreptococcaceae  Incertae Sedis | 1 | unclassified Clostridia | 1 |
| Porphyromonas | 1 | Subdoligranulum | 1 |
| unclassified Bacteroidales | 1 | unclassified Coriobacteriaceae | 1 |
| Capnocytophaga | 1 | Ruminococcaceae Incertae Sedis | 1 |
| unclassified Prevotellaceae | 1 | Clostridium | 1 |

The relative abundance (in percentage) of the 20 most dominant taxonomic groups found throat and fecal samples, respectively. The abundance of all OTUs belonging to the same genus, or were unclassified at the same taxonomic level, were summed.
