# Supplementary material for: Short-Term Antibiotic Treatment Has Differing Long-Term Impacts on the Human Throat and Gut Microbiome
Source: PLoS One. 2010 Mar 24;5(3):e9836. doi: 10.1371/journal.pone.0009836 (PMC2844414; doi:10.1371/journal.pone.0009836)
Supplement: Table S5 — Diversity estimations. Shannon diversity index was calculated based on OTU frequency. The diversity index decreased at day 8–13 in both throat- and fecal samples following antibiotic treatment while it remained stable within the controls during the whole time period. (0.04 MB DOC) [file pone.0009836.s011.doc]

Table S5. Diversity estimations.

|  | Controls | | | Patients | | |
| --- | --- | --- | --- | --- | --- | --- |
| Throat | A | B | C | D | E | F |
| Day 0 | 4.04 | 4.20 | 3.20 | 3.00 | 3.51 | 4.27 |
| Day 8-13 | 4.12 | 4.08 | 2.94 | 2.95 | 1.22 | 3.22 |
| 1 year | 4.27 | 3.83 | 4.03 | 3.39 | 2.86 | 3.78 |
| 4 years | 3.08 | 3.93 | 3.23 | 3.01 | 2.02 | 3.32 |
|  | Controls | | | Patients | | |
| Feces | A | B | C | D | E | F |
| Day 0 | 3.89 | 4.76 | 4.09 | 3.83 | 4.88 | 3.89 |
| Day 8-13 | 4.02 | 4.79 | 3.80 | 2.94 | 3.46 | 2.45 |
| 1 year | 4.46 | 4.16 | 4.04 | 4.52 | 4.94 | 4.07 |
| 4 years | 5.32 | 4.72 | 4.62 | 5.10 | 4.46 | 3.55 |

Shannon diversity index was calculated based on OTU frequency. The diversity index decreased at day 8-13 in both throat- and fecal samples following antibiotic treatment while it remained stable within the controls during the whole time period.
