# Supplementary material for: Short-Term Antibiotic Treatment Has Differing Long-Term Impacts on the Human Throat and Gut Microbiome
Source: PLoS One. 2010 Mar 24;5(3):e9836. doi: 10.1371/journal.pone.0009836 (PMC2844414; doi:10.1371/journal.pone.0009836)
Supplement: Table S6 — Features of participants included in the study. (0.03 MB DOC) [file pone.0009836.s012.doc]

Table S6. Features of participants included in the study.

| Subject | Gender | Age (years) |
| --- | --- | --- |
| Control A | Male | 70 |
| Control B | Female | 71 |
| Control C | Female | 75 |
| Patient D | Male | 52 |
| Patient E | Female | 57 |
| Patient F | Female | 72 |
