# Supplementary material for: Short-Term Antibiotic Treatment Has Differing Long-Term Impacts on the Human Throat and Gut Microbiome
Source: PLoS One. 2010 Mar 24;5(3):e9836. doi: 10.1371/journal.pone.0009836 (PMC2844414; doi:10.1371/journal.pone.0009836)
Supplement: Table S7 — Oligonucleotides, adaptor, and sample specific barcode sequences for 16S rRNA sequencing. (0.03 MB DOC) [file pone.0009836.s013.doc]

Table S7. Oligonucleotides, adaptor, and sample specific barcode sequences for 16S rRNA sequencing.

| Primer name | Adaptor sequence 5’-3’ | Barcode sequence 5’-3’ | Primer sequence 5’-3’ | Reference |
| --- | --- | --- | --- | --- |
| 784f | GCCTTGCCAGCCCGCTCAG |  | AGGATTAGATACCCTGGTA | (24) |
| 1061r | GCCTCCCTCGCGCCATCAG | CGAT | CRRCACGAGCTGACGAC | (24) |
| 1061r | GCCTCCCTCGCGCCATCAG | CATG | CRRCACGAGCTGACGAC | (24) |
| 1061r | GCCTCCCTCGCGCCATCAG | CTGA | CRRCACGAGCTGACGAC | (24) |
| 1061r | GCCTCCCTCGCGCCATCAG | CGTA | CRRCACGAGCTGACGAC | (24) |
