# Supplementary material for: Short-Term Antibiotic Treatment Has Differing Long-Term Impacts on the Human Throat and Gut Microbiome
Source: PLoS One. 2010 Mar 24;5(3):e9836. doi: 10.1371/journal.pone.0009836 (PMC2844414; doi:10.1371/journal.pone.0009836)
Supplement: Table S8 — Oligonucleotides for terminal-restriction fragment length polymorphism and real-time PCR. (0.03 MB DOC) [file pone.0009836.s014.doc]

Table S8. Oligonucleotides for terminal-restriction fragment length polymorphism and real-time PCR.

| Target gene | Primer sequence 5’-3’ | Reference |
| --- | --- | --- |
| 16S rRNA gene (T-RFLP) | F: AGAGTTTGATCCTGGCTCAG | (43) |
|  | R: CCGTCAATTCCTTTRAGTTT | (44) |
| *erm* gene (real-time PCR) | F: GTACCTTGGATATTCACCGAACACT | This study |
| *erm* gene (real-time PCR) | R: CATTCCGCTGGCAGCTTAA | This study |
| *erm* gene (real-time PCR) | Probe: TTGCACACTCAAGTCTCGATTCAGCAATTG | This study |
| 16S rRNA gene (real-time PCR) | F: AGAGTTTGATCCTGGCTCAGATTG | This study |
| 16S rRNA gene (real-time PCR) | R: GTTACCGTTCGACTTGCATGTG | This study |
| 16S rRNA gene (real-time PCR) | Probe: ACGCTGGCGGCAGGCCT | This study |
